# Spatio-temporal characteristics of urban air pollutions and their causal relationships: Evidence from Beijing and its neighboring cities

**DOI:** 10.1038/s41598-017-18107-1

**Published:** 2018-01-19

**Authors:** Lei Jiang, Ling Bai

**Affiliations:** 1 College of Economics and Management, Nanjing University of Aeronautics and Astronautics, Nanjing, China; 2 School of Economics, Zhejiang University of Finance and Economics, Hangzhou, China; 3 School of Economics and Management, Nanchang University, Nanchang, China

## Abstract

China has been suffering from serious air pollution for years in response to the rapid industrialization and urbanization. Notably Beijing is one of the most polluted capitals in the world. Hence, the focus of the study area is on Beijing. In the first stage, we analyze spatial and temporal characteristics of air pollution of the 6 cities while in the second stage the Granger causality test is applied to investigate whether air pollution of a city is affected by its neighbors, and vice versa. The findings are the following. Overall, AQI values are high in winter and early spring while low in summer and autumn. Among the 6 cities, Baoding is the major contributor to air pollution in this entire area. Besides, Granger causality test results show that there is a unidirectional relationship running from Baoding to Beijing and a bidirectional relationship between Beijing and Tianjin. In other words, apart from local air pollutants, for example, exhaust gas, air quality of Beijing is affected by air pollution of Tianjin, and vice versa. However, regarding the relationship between Beijing and Baoding, air quality of Beijing is just affected by air pollution of Baoding, since Baoding is much polluted than Beijing.

## Introduction

China, the largest developing country, has been suffering from severe air pollution for years as a result of the rapid economic growth, the large scale of industrialization and urbanization^1,2^. A large number of Chinese people is at risk of air pollution, since China is the most populous country all over the world. In particular, in 2013 January the North China experienced severe and persistent haze pollution attack. This extreme haze event influenced 800 million people, covering 1.4 million square kilometers of China^3^. At present, large parts of China are frequently affected by air pollution events, notably in the economically developed regions, such as the North China Plain, the Yangtze River Delta, and the Pearl River Delta^4,5^. The frequent air pollution is one of the top environmental concerns. It has posed a huge threaten to health, and even life. Furthermore, air pollution has become the fourth primary risk factor in all deaths after heart attack, dietary risk and smoking in China according to latest statistics^6,7^. Moreover, it is able to cause other diseases, for example, reversible respiratory problems, asthma, lung and heart failure-related mortality^8,9^. Consequently, air quality degradation in Chinese cities has attracted great attention in recent years^10^.

In a bid to address the severe issue of air pollution, a growing number of studies on various primary air pollutants has surged in recent years, for example, total particulate matter. According to the official statistics by the Ministry of Environmental Protection of China, approximately 85–90% of the primary air pollutants in most Chinese cities are particulate matter^11^, notably PM_2.5_ and PM_10_. A recent study by Chen *et al*.^12^ showed that long-term exposure to high particulate matter might reduce life expectancy by about 3 years. PM_2.5_ is particulate matter with an aerodynamic diameter less than 2.5 μm. High PM_2.5_ concentrations not only pose a huge threat to human health^13,14^, but also impair visibility^15^, even leading to traffic accidents. Hence, forecast of PM_2.5_ levels is of vital importance for issuing pollution alerts that inform the public to reduce exposure to high PM_2.5_ concentrations and restrict outdoor activities. For instance, Zhou *et al*.^16^ applied ensemble empirical mode decomposition method and regression neural network to predict PM_2.5_ concentration based on a time series data set of Xi’an China from January 1 to November 1 in 2013. The accurate results were obtained by using the hybrid model. Besides, Fang *et al*.^17^ investigated spatio-temporal characteristics of PM_2.5_ in China by using observed data from 945 monitoring sites in 2014. They found that PM_2.5_ concentrations showed significant seasonal variation. Specifically, it is high in autumn and winter while low in spring and summer. Moreover, PM_2.5_ concentrations presented distinct spatial agglomeration. Elser *et al*.^2^ analyzed PM_2.5_ chemical composition and sources in major cities, namely Beijing and Xi’an in China from the perspective of atmospheric chemistry and physics based on a data collected when extreme haze events occurred during winter in 2013–2014. In addition to PM_2.5_, a large number of studies have also concentrated on PM_10_ in China, notably several Chinese cities^18,19^, for example, Beijing^20,21^, Shanghai^22,23^, Nanjing^24^, or urban clusters, for example, the Yangtze River Delta^25,26^, and the Pearl River Delta Region^27^. According to official statistics, very few large cities in China meet environmental standards according to the World Health Organization.

The North China Plain is frequently attacked by heavy air pollution every winter and early spring, which is home to several of the world’s most polluted cities^28^. More importantly, Beijing, the capital of China, is located in the plain. It has long been one of the most polluted capitals in the world^29^, attracting great attention from the Chinese central government and the public^30^, because it is a mega city with a population of 21.7 millions in 2015. Consequently, the mounting complaints about air quality from the public have been rising for years. Hence, a lot of studies have dedicated to analyzing the severe issue of air pollution in the region. Similarly, they have placed emphasis on the atmospheric chemistry and physics of air pollutants, for example, PM_2.5_ and PM_10_, and meteorological factors^31^.

The above-mentioned air pollutants, namely, particulate matter are main causes for air quality degradation. The serious issue of air pollution in China has triggered public anxiety and has attracted official concerns. The Chinese central government has been aware of air pollution for almost four decades. From 1998, it started to report the weekly air pollution index (API), which takes into account three pollutants, namely, total suspend particle, nitrogen oxide, and sulfur dioxide^32^. However, it was found that daily API values were often less than PM_10_ concentrations. Moreover, the frequent haze-fog event contradicts public perception of air pollution, which has forced the Ministry of Environmental Protection of China to release a new national ambient air quality evaluation standard^33^.

In order to improve air quality evaluation index, an accurate, comprehensive and dimensionless indicator for air pollution, viz. air quality index (AQI) was developed by the Ministry of Environmental Protection of China with PM_2.5_, PM_10_, ozone, and carbon monoxide added to the new index, on the basis of the United States Environmental Protection Agency AQI^34,35^. Since then, the old API has been replaced by the new AQI evaluation standard, since AQI is a better indicator of local air quality^27^.

Basically, AQI has already served as a guide for the governments to inform the public to take protection measures in a bid to avoid long-time exposure to air pollution^36^. Subsequently, it has gain popularity in measuring air quality among the public and the academic circle. A growing number of literature on AQI in China has surged in recent years. For example, Jiang *et al*.^37^ analyzed the relationship between AQI published officially and air pollution posted in Chinese tweeter by using a new social media framework. They found that the filtered social media messages were closely correlated with AQI. Interestingly, Li and Peng^38^ from the perspective of behavioral finance investigated the effect of air pollution on stock returns of depressed moods, since environmental stimuli, for example, air pollution, sunshine, can be used as proxies for collective mood swings. They found that air pollution was a behavioral factor with some connection to stock returns in China. Lin and Wang^39^ analyzed the driving factors of air pollution by using AQI data of 74 Chinese cities in 2014. They drew the conclusion that energy consumption, industrialization and technological progress were attributed to reduced air quality while economic development was a contributor to the improvement of air quality. Besides, a large number of prior works have focused on the spatio-temporal distribution of AQI in some specific Chinese cities, for example, Bejing^40^.

The focus of this study is also on air pollution of Beijing. Generally, Beijing suffers from multiple air pollutants, including sulfur dioxide, nitrogen dioxide, ozone, and particulate matter^29^. Hence, one single pollutant cannot reflect how polluted Beijing is. Since AQI value is determined by these mentioned-above criteria pollutants, it is a best comprehensive index to exactly measure air quality. Besides, one possible shortcoming of these prior works is that they ignore air pollutants from other cities when they analyze air pollution of one city. In other words, air quality of a city is not only affected by its local air pollutants, but also by air pollutants diffused from neighboring cities, since air pollutants disperse across borders. To sum up, air quality of a city tends to be affected by its polluted neighbors.

Many researchers also focused on air pollution of Beijing metropolitan area or Beijing–Tianjin–Hebei region since it is one of the most heavily polluted regions in China. For example, Li *et al*.^41^ applied source apportionment and source sensitivity methods to identify source regions for PM_2.5_ exposure in Beijing–Tianjin–Hebei region in 2013. Li *et al*.^42^ used the WRF-CHEM model to evaluate the contributions of trans-boundary transport to the air quality in Beijing. These aforementioned studies tend to employ atmosphere simulation method since it can clearly involve the effect of climate conditions and provide more details about the mechanisms of transboundary air pollution of the Beijing metropolitan area. However, in this study we aim to provide a novel insight into trans-boundary air pollution of Beijing and its neighbors from the econometric point of view. Its advantages lie in two aspects. One is that the econometric model, specifically Granger causality test, avoids to taking into account the complicated dynamic mechanism in the trans-boundary air pollution. If the current air quality of y city can be explained by past air quality of its neighbor x city, it implies that air pollution of x city causes air pollution of y city. The other is that Granger causality test requires a smaller data size than simulation model, but has a strong econometric explanation that can disclose how air pollution of cities affects each other.

Hence, the main objectives of this study lie in two aspects. One is to analyze spatial and temporal characteristics of AQI values of Beijing and its neighboring cities. This is because understanding spatial distribution and temporal variation of air pollution contributes to implementing city targeted and effective policies to mitigate air pollutants in the most polluted cities when meteorological conditions are the worst. The other one is to investigate how air pollution of these cities interacts by means of an econometric method, different from prior works. In other words, this study attempts to disclose if air quality of a city is affected by its neighbors, or vice versa. Moreover, it may provide a novel insight into how air quality of Beijing is affected and shed light on the mechanism from the viewpoint of empirical econometrics. Besides, the findings may help policy-makers effectively and efficiently predict and control air pollution of the most polluted city, since it may be the major contributor to air pollution of the entire area. To sum up, this research is of great significance to pave a possible way for the solution to air pollution of Beijing.

The rest of this paper is organized as follows. Section 2 introduces methods used in this study and data sources. Section 3 presents spatial and temporal characteristics of AQI and discusses empirical results. Section 4 concludes.

## Methods and Data Sources

One aim of this study is to test for causal relationships between air pollution of Beijing and its neighboring cities by means of a time-series econometric method, specifically, Granger causality test. However, the first step is to test if all AQI values are stationary and integrated of the same order. Hence, a unit root test, namely, augmented Dickey Fuller (ADF) test is first introduced, followed by Granger causality test. Then, the data sources are given.

### Unit root test

Before testing for causal relationships of air pollution of these cities, the first step is to test if all variables are stationary and integrated of the same order. Hence, a unit root test is needed to test if AQI of each city is a stationary time series.

In this study we employ ADF unit root test proposed by Dickey and Fuller^43,44^, which is most widely applied in empirical studies. The null hypothesis of the ADF test is that “the time series has a unit root”. If the null hypothesis is rejected, the series is a stationary time series, or said to be integrated of order zero, or I(0) for short. On the other hand, if the null cannot be rejected, and the first difference of the series is stationary, the series is said to be integrated of order one, or I(1).

The ADF test has three types of assumptions, namely, no intercept and no trend (Equation 1), intercept (Equation 2), and intercept and trend (Equation 3).

1 $${\rm{\Delta }}{y}_{t}=\theta {y}_{t-1}+\sum _{i=1}^{p}{\lambda }_{i}{\rm{\Delta }}{y}_{t-i}+{\mu }_{i}$$

where *y*_*t*_ is the series in time t; *δ* denotes the first difference; *μ* is the error term with a mean 0 and a variance *σ*^2^.

2 $${\rm{\Delta }}{y}_{t}=\alpha +\theta {y}_{t-1}+\sum _{i=1}^{p}{\lambda }_{i}{\rm{\Delta }}{y}_{t-i}+{\mu }_{i}$$

where *α* denotes the intercept term.

3 $${\rm{\Delta }}{y}_{t}=\alpha +\beta t+\theta {y}_{t-1}+\sum _{i=1}^{p}{\lambda }_{i}{\rm{\Delta }}{y}_{t-i}+{\mu }_{i}$$

where *β* denotes the time trend. The null hypothesis of the ADF test is H_0_: *θ* = 0, and the alternative hypothesis is H_1_: *θ* < 0.

### Granger causality test

Granger causality test, firstly proposed by Granger^45^, is a commonly-used test to investigate causal relationships between two variables. It is a statistical hypothesis test for the question of if one variable affects the other. Technically speaking, *x* and *y* are two time series. If “*x* causes *y*” by means of a set of statistics, it indicates that the current *y* can be explained by past values of *x* and that adding lagged values of *x* can enhance the explanation. When it comes to air pollution, air quality of a city may be affected not only by the past air quality, but also by air pollution of its neighbors. Hence, in this study it is applied to test for if air pollution of one city affects the other, and vice versa. The Granger causality test model reads as follows.

4 $${y}_{t}={\alpha }_{0}+\sum _{j=1}^{p}{\alpha }_{i}{y}_{t-j}+\sum _{j=1}^{p}{\beta }_{i}{x}_{t-j}+{\varepsilon }_{t}$$

5 $${x}_{t}={\alpha }_{0}+\sum _{j=1}^{p}{\alpha }_{i}{x}_{t-j}+\sum _{j=1}^{p}{\beta }_{i}{y}_{t-j}+{\varepsilon }_{t}$$

The null hypothesis is that “*x* does not Granger cause *y*” in the first regression. Similarly, the null hypothesis of the second equation is that “*y* does not Granger cause *x*”.

Technically speaking, F-statistics are the Wald statistics for the joint hypothesis. The null joint hypothesis is given below.

6 $${H}_{0}:{\beta }_{1}={\beta }_{2}=\cdots ={\beta }_{p}=0$$

On the other hand, the alternative hypothesis is that at least one estimated parameter is not zero. It can be given as follows.

7 $${H}_{1}:\mathrm{At}\,{\rm{least}}\,{\rm{one}}\,{\beta }_{j}\ne 0$$

### Study area and data sources

The focus of the study area is on Beijing and its neighboring cities, part of Jing-Jin-Ji region. The Jing-Jin-Ji region covers two municipalities, namely, Beijing (Jing for short) and Tianjin (Jin for short), and a province, namely, Hebei province (Ji for short). The region had a population of more than 110 million in 2015 that accounted for about 8.1% of China’s total. It is located in the North China Plain, one of the China’s largest metropolitan areas. It has an area of 216, 600 square kilometers, about 2.25% of the entire nation.

The Jing-Jin-Ji region is an economically developed metropolitan area in China with a high economic growth rate. Its gross regional product (GRP) amounted to 6935.89 billion Yuan in 2015. It has been playing an important role in China’s economy. Specifically, its GRP accounted for more than 10% of China’s GDP in 2015.

On the other hand, it has also been suffering from the serious issue of environmental pollution, notably air pollution for years, due to its rapid industrialization and urbanization. For example, industrial sulfur dioxide emissions amounted to 1.365 million tons in 2015, accounting for 7.3% of the China’s total. It is attributed to a large deal of energy consumed. For example, it consumed 443.26 billion tons of standard coal equivalent (SCE), accounting for 10% of the total energy consumption of China. It is worth noting that Hebei province in the region is well-known for its steel industry. The steel production of Hebei province in 2015 amounted to more than 250 million tons, accounting for almost one quarter of China’s total output. The single industrial sector emitted quite a lot of pollutants, contributing to air quality degradation as a result of more than 100 millions tons SCE consumed in 2015. It has been the main reason for air pollution in this region.

**Figure 1:**
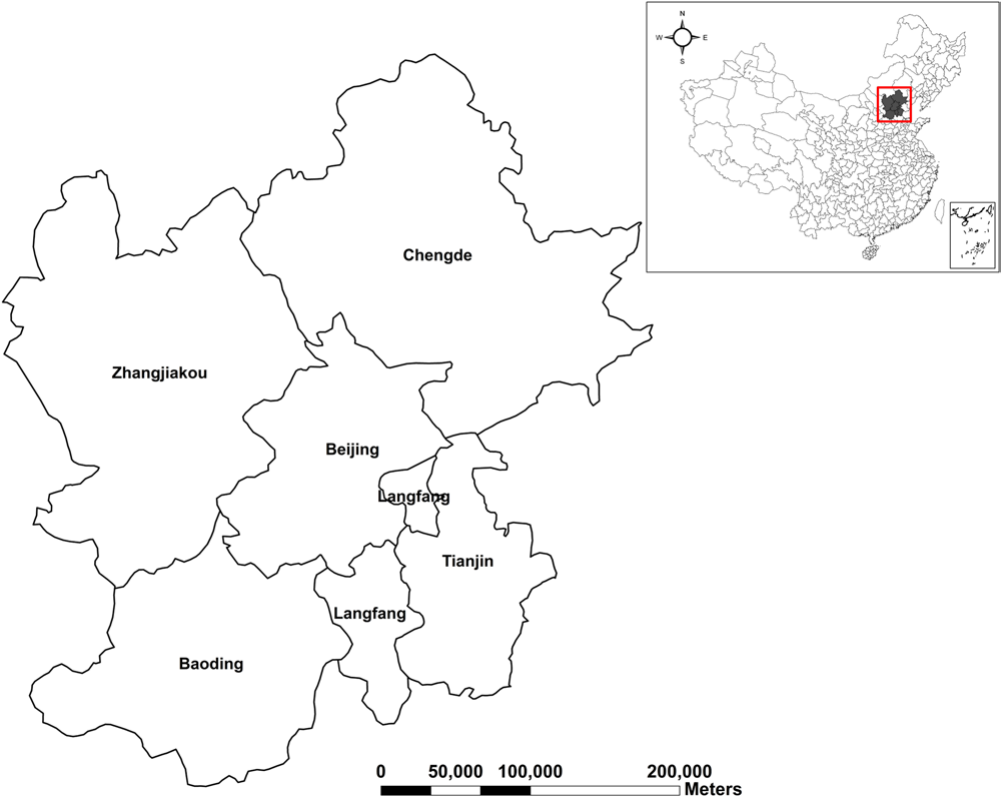
Map of study area. The map was generated using ArcGIS (Version 10.3.1., https://www.arcgis.com). The source of the map is GADM database of Global Administrative Areas (http://www.gadm.org/). The study area is part of the map of China.

Beijing is one of the most polluted capitals all over the world. Apart from its traffic exhaust gas, another main reason is that it is surrounded by the polluted neighbors. In a bid to solve the serious problem of air pollution, it is of great significance to research on if air quality of Beijing is affected by its neighbors. Hence, we select its 5 neighboring cities, viz., Tianjin, Baoding, Zhangjiakou, Chengde, Langfang. In other words, Beijing is surrounded by the 5 cites. The study area is shown in Fig. 1.

AQI level is determined by six criteria pollutants, namely, SO_2_, NO_2_, CO, O_3_, PM_2.5_, and PM_10_ according to the Ambient Air Quality Index (AQI) Technical Provisions issued by the Ministry of Environmental Protection of China. Data for AQI are obtained from the national platform of the Ministry of Environmental Protection (http://106.37.208.233:20035/ (in Chinese)). The time series data are from January 1 to December 31 in 2016. In other words, there are 366 observations. The descriptive statistics for AQI values of the 6 cities (means, standard deviations (S.D), minimum (Min) and maximum (Max) values) are presented in Table 1.

**Table 1:** Descriptive statistics of AQI.

City	Mean	S.D.	Min	Max	Excellence Rate
Beijing	104.49	70.18	24.29	429.96	58.47%
Tianjin	101.99	59.74	31.00	389.29	63.11%
Baoding	128.34	76.10	29.67	438.46	44.26%
Zhangjiakou	71.63	30.71	28.83	236.39	85.25%
Chengde	71.55	33.89	23.08	248.50	83.06%
Langfang	100.66	64.23	28.38	431.25	62.02%

## Empirical Results and Discussions

The empirical analysis section consists of two stages. In the first stage we present spatial and temporal variation of AQI values of the 6 cities in order to understand how air pollution changes during the whole year, and then in the second stage causal relationships result.

### Temporal variation analysis

**Figure 2:**
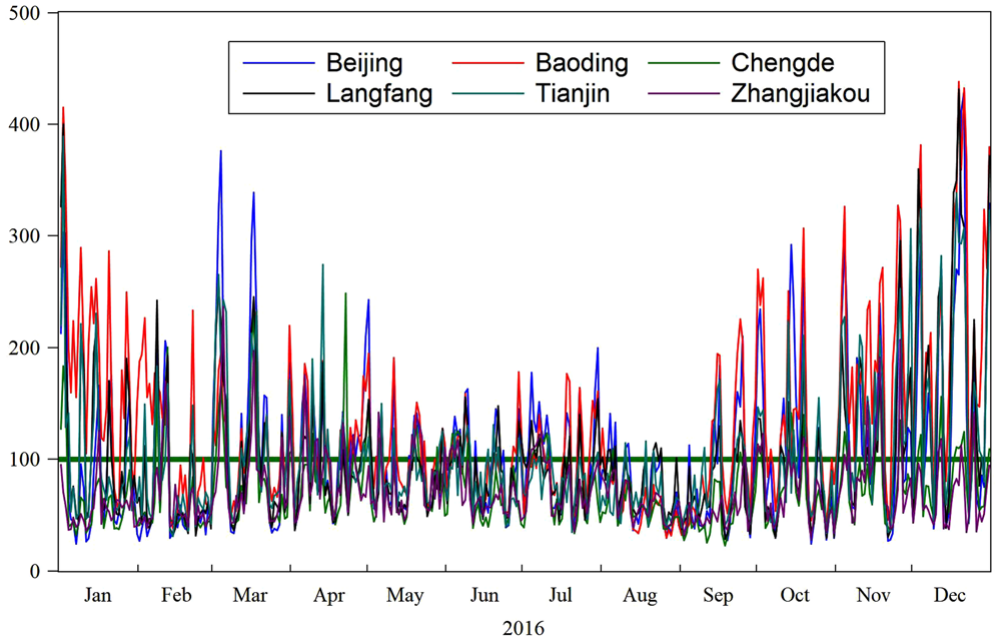
Daily AQI values of the 6 cities in 2016.

We simply plot time series of AQI of each city in order to explicitly disclose their fluctuation patterns. It is shown in Fig. 2.

Figure 2 displays the panorama of the time series dataset. Specifically, it shows daily AQI values of the 6 cities from January 1 to December 31 in 2016. Note that there is a horizontal bar displayed in Fig. 2 where AQI value in the y-axis is equal to 100, implying it is a critical criterion value. According to the Ambient Air Quality Index (AQI) Technical Provisions (Trial), if the observed value of AQI is less than 100, it applies that the city’s air quality is good, and otherwise polluted. Hence, there is a formula to calculate the excellence rate of air quality, that is, the ratio of the number of days with AQI value less than 100 to the whole days of the year. Also, the AQI level criterion and air pollution level by city are given in Table 2.

**Table 2:** Air pollution level by city (Unit: %).

AQI	Level	Beijing	Tianjin	Baoding	Zhangjiakou	Chengde	Langfang
0–50	Excellent	23.22	10.38	7.92	25.41	32.24	14.75
51–100	Good	35.25	52.73	36.34	59.84	50.82	47.27
101–150	Lightly polluted	21.58	21.86	25.96	12.57	13.39	23.22
151–200	Moderately polluted	10.38	6.28	15.30	1.64	2.46	7.10
201–300	Heavily polluted	7.10	7.10	10.11	0.55	1.09	4.92
300+	Severely polluted	2.46	1.64	4.37	0.00	0.00	2.73

**Figure 3:**
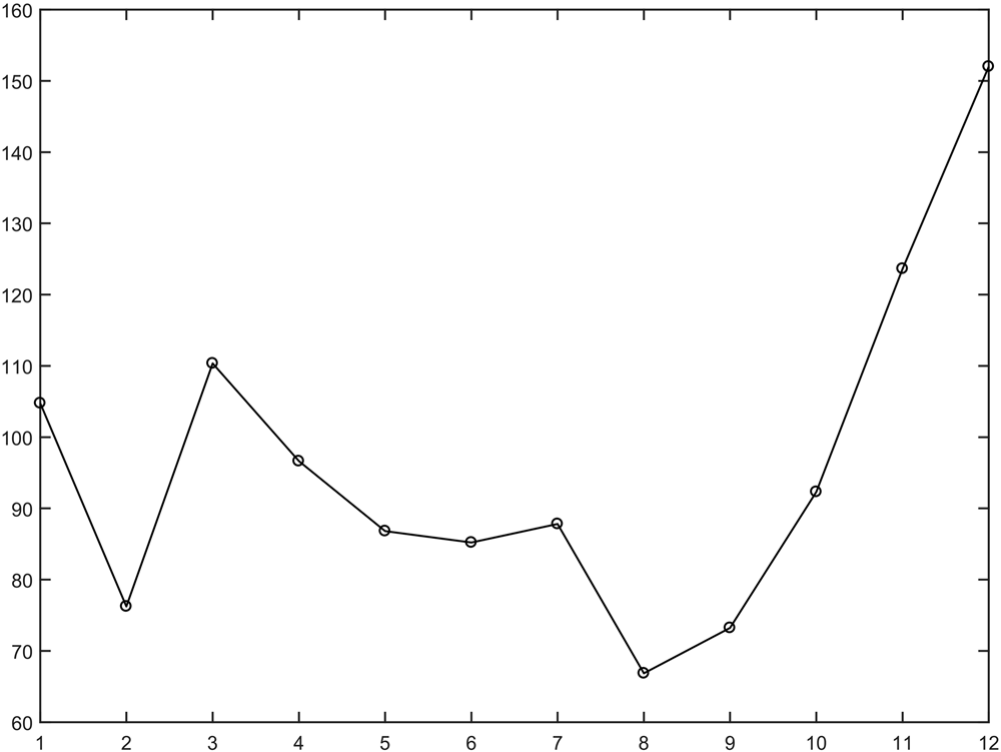
Monthly average AQI values from January to December in 2016.

In order to better understand how AQI values change during the whole year, the monthly average AQI values from January to December are given in Fig. 3. We find that it presents a W-shaped curve. Specifically, it went down from January to February, and then increased to March, and declined to August, and finally continued to increase till December. To sum up, in summer and autumn, AQI values are low, indicating good air quality while in winter and early spring AQI values are high, implying serious air pollution. One biggest possible explanation is that central heating service is provided in winter and early spring in these cities, heavily depending on a large deal of coal combustion. Consequently, various pollutants degrade air quality.

In North China, heating is heavily dependent on coal combustion and approximately 40% of air pollutants comes from coal dust^46^. In particular, central heating service is commonly offered in winter and early spring when the meteorological conditions are just the worst during the whole year. As a consequence, AQI values are usually the highest from November to next January, even to March, since the central heating service is provided from November to next March every year. It should be noted that the entire China is also heavily dependent on fossil fuel, notably coal, which has acted as the major contributor to air quality degradation^47^.

### Spatial distribution analysis

**Figure 4:**
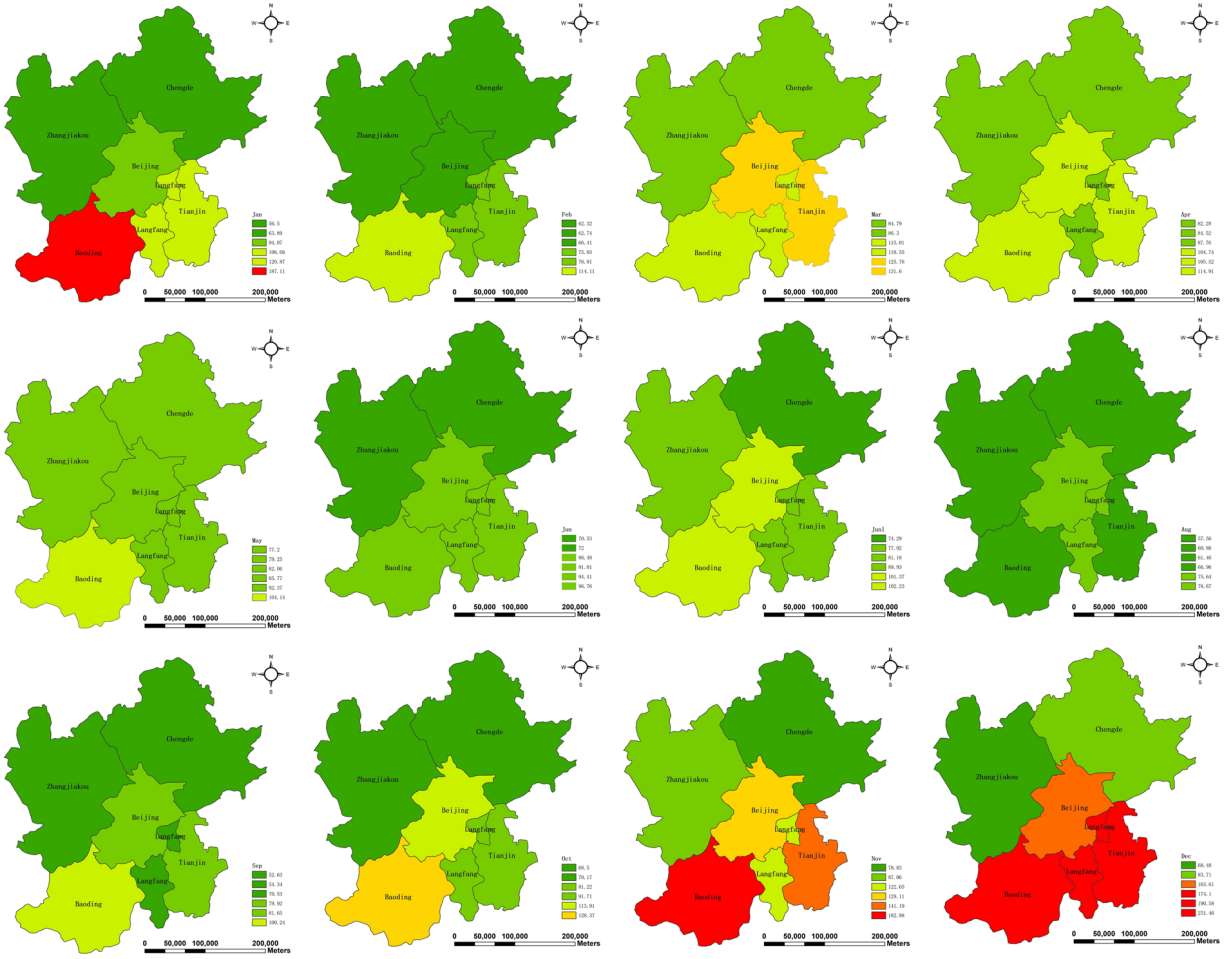
Spatial distribution of monthly average values of AQI. The maps were generated using ArcGIS (Version 10.3.1., https://www.arcgis.com). The source of the map is GADM database of Global Administrative Areas (http://www.gadm.org/). The study area is part of the map of China.

We next turn to the spatial distribution analysis in a bid to investigate where air pollution occurs and how it changes. We apply the geovisualization technique to map the monthly average AQI values of the 6 cities from January to December. From Fig. 4, we find that Changde and Zhangjiakou have good air quality throughout the whole year. In January, November and December, Baoding’s air quality is highly polluted. Similarly, in winter Baoding’s close neighboring cities, namely, Beijing, Langfang and Tianjin also face serious air pollution.

**Figure 5:**
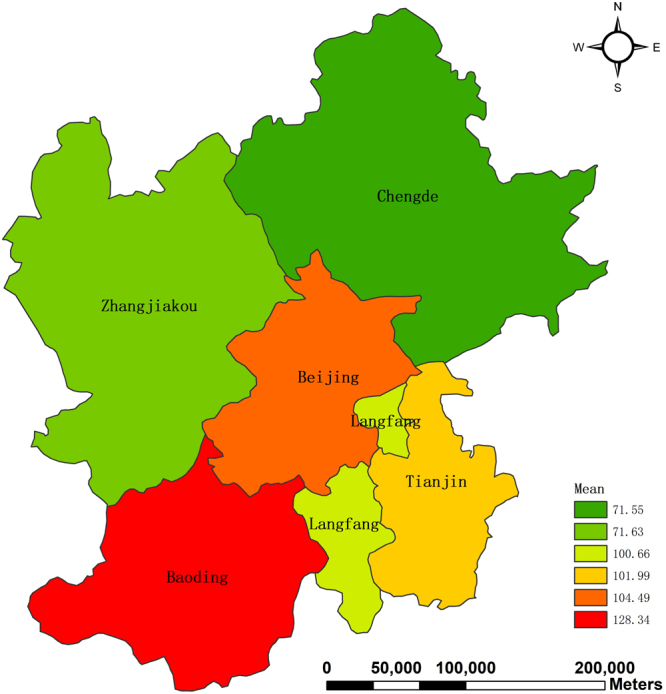
Spatial distribution of excellence rate of air quality. The map was generated using ArcGIS (Version 10.3.1., https://www.arcgis.com). The source of the map is GADM database of Global Administrative Areas (http://www.gadm.org/). The study area is part of the map of China.

In order to explicitly disclose air quality of each city in space, the spatial distribution of the excellence rate of air quality of the 6 cities is given in Fig. 5. From Fig. 5, We find that Baoding has the lowest excellence rate (44.26), indicating that it is the most polluted city among these 6 cities. On the other hand, Zhangjiakou has the highest excellence rate (85.25), implying that its air quality is the best. Notably the rate of Beijing amounts to 62.02, suggesting that during the whole year 2016 the number of days with AQI values less than 100 is more than a half year.

### Granger causality test results

Figure 5 displays that AQI values of each city seems to fluctuate up and down during the whole year. In other words, these time series present a pattern with an intercept. Hence, the intercept term should be taken into account when testing for unit roots. The results of the ADF test are shown in Table 3.

**Table 3:** Unit root test results.

City	Intercept	Intercept and trend
Beijing	−11.355	−11.763
Tianjin	−10.387	−10.686
Baoding	−7.962	−8.149
Zhangjiakou	−11.187	−11.173
Chengde	−11.324	−11.314
Langfang	−10.468	−10.886
1% level	−3.448	−3.983
5% level	−2.869	−3.422
10% level	−2.571	−3.134

In Table 3 t statistics are reported, followed by the critical values for the t test at the 1%, 5%, and 10% significance level. In the second column it can be found that t statistics for AQI time series of each city is far less than the critical value at the 1% significance level (Note: t-statistic is negative.). In other words, the null hypothesis is strongly rejected, indicating that each time series is I(0). For robustness, the results of the ADF test with a trend and an intercept are also reported in the third column of Table 3. Similarly, the null hypothesis is also strongly rejected. To sum up, AQI time series of each city has no unit root, or I(0).

After unit root tests, naturally the next step is to conduct the cointegration test. An easy way to test for the cointegration relationships between two cities is to perform the Engle-Granger two-step method. Since these time series are I(0), linear combination of them must be stationary. In other words, the residual series for the cointegration regression is likely stationary. Hence, the cointegation test may not work well. On the other hand, in this research we pay only attention to the causal relationships between two cities which have common borders. In other words, we are incapable of taking these 6 cities as an integrated region to perform the cointegration test. This is because the mechanism of air pollution may be complicated and even inexplainable by these time series data. In addition, it is also beyond the scope of this research. The main aim of this research is to explore the causal relationships between the time series data from the viewpoint of the econometric method.

**Figure 6:**
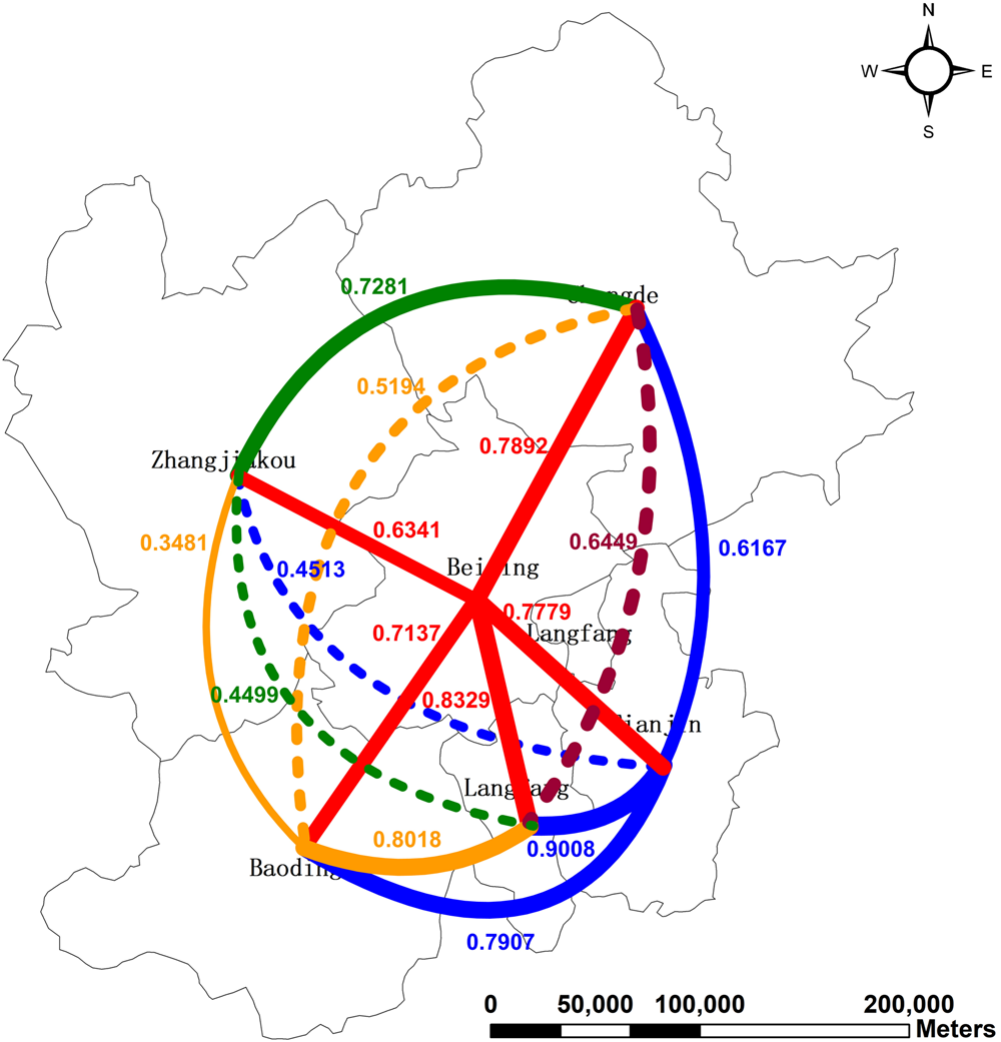
Correlation relationships between cities. The map was generated using ArcGIS (Version 10.3.1., https://www.arcgis.com). The source of the map is GADM database of Global Administrative Areas (http://www.gadm.org/). The study area is part of the map of China. Note: Solid lines imply that two cities have common borders while dash lines imply two cities without common borders.

Before causal relationships, we turn our eyes to the correlation relationships between AQI values of cities. The Pearson correlation coefficient test is then performed. In order to explicitly display how close these time series are, the correlation coefficients and their magnitude are plotted in Table 4 and Fig. 6. Note that all coefficients are significant and positive.

**Table 4:** Pearson correlation coefficients between cities.

City	Beijing	Tianjin	Baoding	Zhangjiakou	Chengde	Langfang
Beijing	1.000					
Tianjin	0.778	1.000				
Baoding	0.714	0.791	1.000			
Zhangjiakou	0.634	0.451	0.348	1.000		
Chengde	0.789	0.617	0.519	0.728	1.000	
Langfang	0.833	0.901	0.802	0.450	0.645	1.000

The second column of Table 4 reports the Pearson correlation coefficients between Beijing and its neighbors, namely, Tianjin, Baoding, Zhangjiakou, Chengde, and Langfang, implying that how closely related these time series are. Technically, the high coefficient value, the more related. It can be found that these coefficients are larger than 0.6, indicating that Beijing’s AQI is closely related with its neighbors’ AQI values.

More interestingly, for three pairs of cities without common borders, their coefficients are smaller, specifically, Chengde and Baoding (0.5194), Zhangjiakou and Tianjin (0.4513), Zhangjiakou and Langfang (0.4499). Besides, the relationship between Zhangjiakou and Baoding is also not too closed, having a weak coefficient of 0.3481. This is because Baoding is seriously polluted with high AQI values throughout the whole year while Zhangjiakou is not, presenting a good environment.

In order to explicitly present the relationships between cities with common borders, we use geovisualization technique to plot the Pearson coefficients and the magnitude. It is presented in Fig. 6.

**Figure 7:**
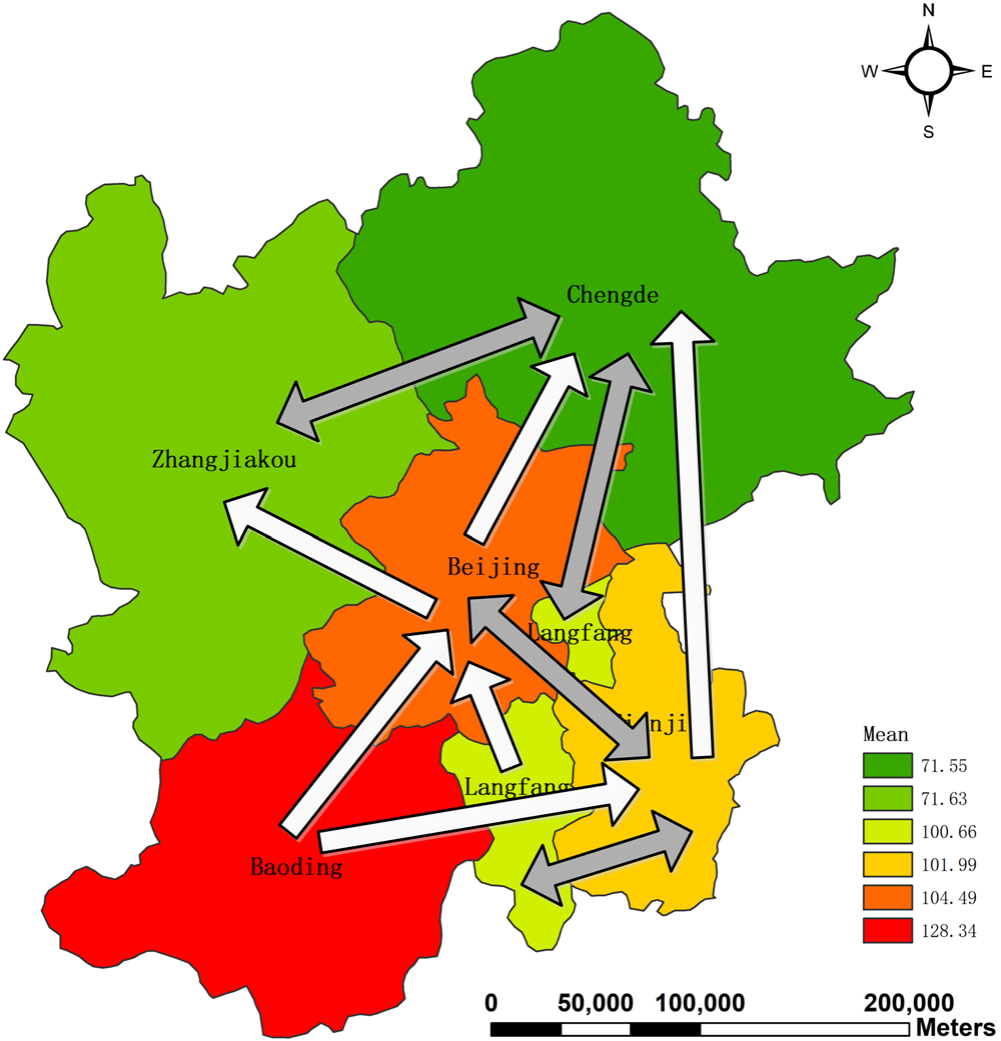
Causality relationships of air pollution between cities. The map was generated using ArcGIS (Version 10.3.1., https://www.arcgis.com). The source of the map is GADM database of Global Administrative Areas (http://www.gadm.org/). The study area is part of the map of China.

Although the Pearson coefficients present a measure of the pairwise correlation between two time series, it is unable to capture which one is affected by the other, or vice versa. What’s more, the strong correlation coefficient does not imply causality. Hence, we use the Granger causality test to discover the causal relationships between cities with and without common borders. In order to better present the causality results, we exhibit a map of causal relationships between cities also by means of employing the geovisualization technique (See Fig. 7). Note that the double-headed arrow represents a bidirectional causal relationship, implying that they are affected by each other. Moreover, the single-headed arrow indicates a unidirectional causal relationship. Besides, to better understand causal relationships, the average value of AQI of each city is also considered and given in the Fig. 7.

As shown in Fig. 7, there are four pairs of bidirectional causal relationships, namely, Beijing and Tianjin, Tianjin and Langfang, Chengde and Langfang, and Zhangjiakou and Chengde. We find that the two cities with bidirectional causal relationships have similar mean values of AQI. For example, Beijing’s AQI has a mean value of 104.49 and Tianjin 101.99. Moreover, the mean values of Zhangjiakou and Chengde are 71.63 and 71.55, respectively. To conclude, two neighboring cities with similar mean values of AQI tend to have bidirectional causal relationships.

We also find 6 unidirectional causal relationships running from Baoding to Beijing, from Baoding to Tianjin, from Beijing to Chengde, from Beijing to Zhangjiakou, from Tianjin to Chengde, from Langfang to Beijing, respectively. Overall, it clearly presents a pollution diffusion pattern, namely, from the highly polluted Baoding to less polluted cities. Our empirical findings have important policy implications for the government. In order to solve the serious issue of air pollution in this area, improvements of air quality and reduction of pollution sources of Baoding is the first priority. To sum up, it follows the conclusions that highly-polluted cities affect ones with low pollution and that for those cities which have similar AQI values, they tend to affect each other.

**Figure 8:**
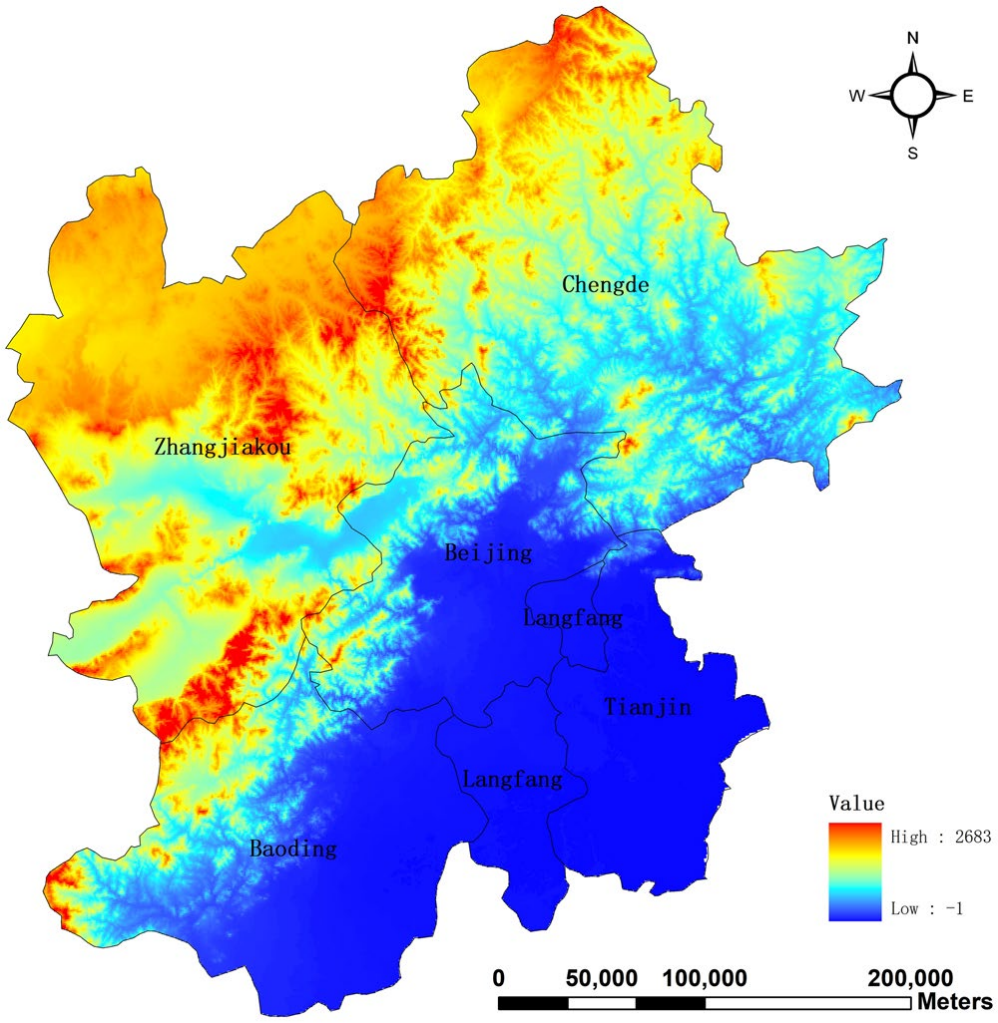
Evaluation of the study area. The map was generated using ArcGIS (Version 10.3.1., https://www.arcgis.com). The source of the map is GADM database of Global Administrative Areas (http://www.gadm.org/). The study area is part of the map of China.

Also, we attempt to give an explanation of how air pollution between cities interacts from the perspective of geography. The terrain’s surface of the study area is shown in Fig. 8. From Fig. 8, it can be found that the study area is separated by the Yanshan Mountain Ridge into two parts. One is the mountainous area with high evaluation, including two cities, namely, Zhangjiakou and Chengde. The rest is the plain area, part of the North China plain, where air pollutants tend to concentrate. This is also the main reason why this area is highly polluted.

On the other hand, highly polluted Beijing affects air quality of Chengde and Zhangjiakou. However, the influence of Beijing on them is relatively limited because they are separated by the Yanshan Mountain Ridge. Moreover, interestingly it can be found that there is no causal relationship between highly-polluted Baoding and Zhangjiakou. Hence, this is the major geography reason why Chengde and Zhangjiakou are not as polluted as Baoding and Beijing.

Besides, we also find that there are no causal relationships between Zhangjiakou and Langfang, and Zhangjiakou and Tianjin. In other words, more polluted Langfang and Tianjin cannot affect air quality of Zhangjiakou, and vice versa. This lies in two possible reasons. One is that Tianjin and Langfang is much far away from Zhangjiakou in geography. The other is that air pollution diffusion is blocked by the Yanshan Mountain Ridge.

## Conclusions and Future Direction

In this paper, in the first stage we spatially and temporally analyzed AQI values of Beijing, Tianjin, Baoding, Zhangjiakou, Chengde, and Langfang in 2016. From the temporal perspective, AQI values are found to be low in summer and autumn, implying high air quality while air quality is the most serious in winter and early spring. From the spatial and temporal perspective, Chengde and Zhangjiakou are found to have good air quality since their AQI values are low throughout the whole year. Moreover, Baoding has the highest AQI values in winter. Among the 6 cities, it is the most polluted. However, in summer Baoding also enjoys good air quality. From the spatial distribution of the excellence rate, it can also be found that Baoding is highly polluted since its excellence rate is the lowest while Zhangjiakou has good air quality throughout the whole year. In the second stage we conducted the Granger causality test to investigate how air pollution of these cities affects each other. The findings are the following. There are bidirectional causal relationships between Beijing and Tianjin, Tianjin and Langfang, Chengde and Langfang, and Zhangjiakou and Chengde. Moreover, 6 unidirectional causality relationships are also found, namely, running from Baoding to Beijing, from Baoding to Tianjin, from Beijing to Chengde, from Beijing to Zhangjiakou, from Tianjin to Chengde, and from Langfang to Beijing. From the above analysis, it follows that Baoding is mainly responsible for air pollution in the area.

Jing-Jin-Ji region is one the most heavily polluted regions in China as a result of rapid economic growth, the large scale of industrialization, and urbanization. China’s income levels have been ever increasing since reforms and opening-up policies. A massive number of energy-intensive products, like cars is consumed that leads to a plenty of pollution emissions and environmental degradation. Hence, environmental protection education and reinforcement environmental awareness are needed to encourage people to adopt a low carbon lifestyle. Besides, the Chinese economy is greatly attributed to the large scale of industrialization. Consequently, the secondary industry has long been the biggest energy consumer and pollution emitter in China. In other words, it should be responsible for ever-deteriorating air quality and environment quality. Hence, the industrial transition moving from the highly-polluted secondary industry to the high-value-added service industry should be encouraged by the Chinese government. Moreover, a series of vital environmental policies are also urgently needed to enhance environmental protection expenditure.

On the other hand, this study also suffers from three shortcomings. One is that we only consider those cities which have common borders with Beijing and ignore other polluted cities in Hebei province, for example, Tangshan, Shijiazhuang, Handan etc, which also may affect air quality of Beijing and other cities. This is because these polluted cities are far away from Beijing in geography that cannot influence Beijing as much as Beijing’s neighboring cities. The second is that the mechanism of air pollution, notably the impacts of meteorological factors, such as wind and relative humidity, is unable to be taken into consideration since it is beyond the scope of the research. We explore how air pollution of these cities affect each other by means of the time series data from the viewpoint of the econometrics. We hope that this research sheds light on the interaction relationships of air pollution between cities from the time series perspective and provide a possible research direction in the future. The last is that the sample data may be limited since it has 366 observations. We in the future hope to enlarge sample size and re-study causal relationships of air pollution of more cities in a bid to obtain robust scientific results.

## References

[1] Zhang XY (2012). Atmospheric aerosol compositions in China: spatial/temporal variability, chemical signature, regional haze distribution and comparisons with global aerosols. Atmos Chem Phys.

[2] Elser M (2016). New insights into PM_2.5_ chemical composition and sources in two major cities in China during extreme haze events using aerosol mass spectrometry. Atmos Chem Phys.

[3] Huang RJ (2014). High secondary aerosol contribution to particulate pollution during haze events in China. Nature.

[4] Hu J, Wang Y, Ying Q, Zhang H (2014). Spatial and temporal variability of PM_2.5_ and PM_10_ over the North China Plain and the Yangtze River Delta, China. Atmos Environ.

[5] Li R (2017). Spatial and temporal variation of particulate matter and gaseous pollutants in China during 2014–2016. Atmos Environ.

[6] Matus K (2012). Health damages from air pollution in China. Global Environ Chang.

[7] Guo H, Wang Y, Zhang H (2017). Characterization of criteria air pollutants in Beijing during 2014–2015. Environ Res.

[8] Phung D (2016). Air pollution and risk of respiratory and cardiovascular hospitalizations in the most populous city in Vietnam. Sci Total Environ.

[9] Tsangari H (2016). Extreme weather and air pollution effects on cardiovascular and respiratory hospital admissions in Cyprus. Sci Total Environ.

[10] Yang YR (2015). Characteristics and formation mechanism of continuous extreme hazes in China: a case study in autumn of 2014 in the north China plain. Atmos Chem Phys.

[11] Wang H (2014). A study of the meteorological causes of a prolonged and severe haze episode in January 2013 over central-eastern China. Atmos Environ.

[12] Chen Y, Ebenstein A, Greenstone M, Li H (2013). Evidence on the impact of sustained exposure to air pollution on life expectancy from China’s Huai River policy. P Natl Acad Sci USA.

[13] Xu H (2016). Inter-annual variability of wintertime PM_2.5_ chemical composition in Xi’an, China: Evidences of changing source emissions. Sci Total Environ.

[14] Tao M (2016). Spatial oscillation of the particle pollution in eastern China during winter: Implications for regional air quality and climate. Atmos Environ.

[15] Fu GQ, Xu WY, Yang RF, Li JB, Zhao CS (2014). The distribution and trends of fog and haze in the North China Plain over the past 30 years. Atmos Chem Phys.

[16] Zhou Q, Jiang H, Wang J, Zhou J (2014). A hybrid model for PM_2.5_ forecasting based on ensemble empirical mode decomposition and a general regression neural network. Sci Total Environ.

[17] Fang C, Wang Z, Xu G (2016). Spatial-temporal characteristics of PM_2.5_ in China: A city-level perspective analysis. J Geogr Sci.

[18] Cheng Z, Jiang J, Fajardo O, Wang S, Hao J (2013). Characteristics and health impacts of particulate matter pollution in China (2001–2011). Atmos Environ.

[19] An X, Hou Q, Li N, Zhai S (2013). Assessment of human exposure level to PM_10_ in China. Atmos Environ.

[20] Li X, Wang Y, Guo X, Wang Y (2013). Seasonal variation and source apportionment of organic and inorganic compounds in PM_2.5_ and PM_10_ particulates in Beijing, China. J Environ Sci-china.

[21] Cao C (2014). Inhalable microorganisms in Beijing’s PM_2.5_ and PM_10_ pollutants during a severe smog event. Environ Sci Technol.

[22] Wang J, Hu Z, Chen Y, Chen Z, Xu S (2013). Contamination characteristics and possible sources of PM_10_ and PM_2.5_ in different functional areas of Shanghai, China. Atmos Environ.

[23] Ma J (2013). Airborne PM_2.5_/PM_10_-associated chlorinated polycyclic aromatic hydrocarbons and their parent compounds in a suburban area in Shanghai, China. Environ Sci Technol.

[24] Shen GF (2014). Ambient levels and temporal variations of PM_2.5_ and PM_10_ at a residential site in the mega-city, Nanjing, in the western Yangtze River Delta, China. J Environ Sci Heal A.

[25] Cheng Z (2014). Impact of biomass burning on haze pollution in the Yangtze River delta, China: a case study in summer 2011. Atmos Chem Phys.

[26] Cheng Z (2013). Long-term trend of haze pollution and impact of particulate matter in the Yangtze River Delta, China. Environ Pollut.

[27] Zheng J, Che W, Zheng Z, Chen L, Zhong L (2013). Analysis of spatial and temporal variability of PM_10_ concentrations using MODIS aerosol optical thickness in the Pearl River Delta Region, China. Aerosol Air Qual Res.

[28] Wang M, Cao C, Li G, Singh RP (2015). Analysis of a severe prolonged regional haze episode in the Yangtze River Delta, China. Atmos Environ.

[29] Hu M, Jia L, Wang J, Pan Y (2013). Spatial and temporal characteristics of particulate matter in Beijing, China using the empirical mode decomposition method. Sci Total Environ.

[30] Guo J (2016). Impact of various emission control schemes on air quality using WRF-CHEM during APEC China 2014. Atmos Environ.

[31] She Q (2017). Air quality and its response to satellite-derived urban form in the Yangtze River Delta, China. Ecol Indic.

[32] Xia TY, Wang JY, Song K, Da LJ (2014). Variations in air quality during rapid urbanization in Shanghai, China. Landsc Ecol Eng.

[33] Chen W, Tang H, Zhao H (2016). Urban air quality evaluations under two versions of the national ambient air quality standards of China. Atmos Pollut Res.

[34] Shen F (2017). Air pollution characteristics and health risks in Henan Province, China. Environ Res.

[35] Liu Y, Wu J, Yu D (2017). Characterizing spatiotemporal patterns of air pollution in China: A multiscale landscape approach. Ecol Indic.

[36] Xu X, You S, Zheng X, Li H (2014). A survey of district heating systems in the heating regions of northern China. Energy.

[37] Jiang W, Wang Y, Tsou MH, Fu X (2015). Using social media to detect outdoor air pollution and monitor air quality index (AQI): a geo-targeted spatiotemporal analysis framework with Sina Weibo (Chinese Twitter). PLOS ONE.

[38] Li Q, Peng CH (2016). The stock market effect of air pollution: evidence from China. Appl. Econ.

[39] Lin X, Wang D (2016). Spatiotemporal evolution of urban air quality and socioeconomic driving forces in China. J Geogr Sci.

[40] Yan S (2016). Spatial and temporal characteristics of air quality and air pollutants in 2013 in Beijing. Environ Sci Pollut R.

[41] Li G, Cao J (2017). Contributions of trans-boundary transport to summertime air quality in Beijing, China. Atmos. Chem. Phys.

[42] Li X (2017). Attribution of PM_2.5_ exposure in Beijing-Tianjin-Hebei region to emissions: Implication to control strategies. Sci Bull.

[43] Dickey DA, Fuller WA (1979). Distribution of the estimators for autoregressive time series with a unit root. J. Amer. Statistical Assoc.

[44] Dickey DA, Fuller WA (1981). Likelihood ratio statistics for autoregressive time series with a unit root. Econometrica.

[45] Granger CW (1969). Investigating causal relations by econometric models and cross-spectral methods. Econometrica.

[46] Li H (2017). Modelling of AQI related to building space heating energy demand based on big data analytics. Appl Energ.

[47] Zheng S, Yi H, Li H (2015). The impacts of provincial energy and environmental policies on air pollution control in China. Renew Sust Energ Rev.

